# Varying temperature and silicon content in nanodiamond growth: effects on silicon-vacancy centres

**DOI:** 10.1038/s41598-018-21953-2

**Published:** 2018-02-28

**Authors:** Sumin Choi, Victor Leong, Valery A. Davydov, Viatcheslav N. Agafonov, Marcus W. O. Cheong, Dmitry A. Kalashnikov, Leonid A. Krivitsky

**Affiliations:** 1 Data Storage Institute, Agency for Science, Technology and Research, 138634 Singapore, Singapore; 2 L.F. Vereshchagin Institute for High Pressure Physics, The Russian Academy of Sciences, Troitsk, Moscow 142190 Russia; 3 GREMAN, UMR CNRS-7347, Université F. Rabelais, 37200 Tours, France

## Abstract

Nanodidamonds containing colour centres open up many applications in quantum information processing, metrology, and quantum sensing. However, controlling the synthesis of nanodiamonds containing silicon vacancy (SiV) centres is still not well understood. Here we study nanodiamonds produced by a high-pressure high-temperature method without catalyst metals, focusing on two samples with clear SiV signatures. Different growth temperatures and relative content of silicon in the initial compound between the samples altered their nanodiamond size distributions and abundance of SiV centres. Our results show that nanodiamond growth can be controlled and optimised for different applications.

## Introduction

Colour centres in diamond have emerged as important quantum emitters for a broad range of applications including bioimaging^1–3^, sensing^4,5^, and quantum nanophotonics^6,7^. One important example is the silicon vacancy (SiV) centre, which has been an active focus of research in recent years due to its attractive optical properties^8–11^, including high brightness, narrow homogenous distribution, stable single photon emission with near-transform-limited linewidths, and minimal spectral diffusion. Its zero-phonon line (ZPL) at 737 nm contains ~70% of the emitted fluorescence, and inversion symmetry grants an insusceptibility to electric field fluctuations. Recent works have also explored the applications of SiV centres based on diamond nanostructures^6,12^.

Nanodiamonds (NDs) containing colour centres can be spatially manipulated and precisely positioned for enhanced coupling to other nanophotonics structures^13,14^ or to fibers^15,16^. The small size of NDs is also advantageous in bioimaging and sensing applications^17,18^, and may enhance coherence times in the SiV centres^19,20^. In principle, the ND composition can be optimised for different applications. For instance, fluorescent imaging probes require a high density of emitters for increased brightness and must be stable against photobleaching, while many quantum networking tasks require single emitters as true single-photon sources.

Here, we explore the ability to control the ND size distribution and abundance of SiV centres by adjusting the growth temperature and relative silicon content in the initial ND growth compound. We perform room-temperature characterisation of several ND samples and compare their physical and optical properties.

## Nanodiamond Preparation

The NDs with SiV centres used in this work were synthesised using a high-pressure high-temperature (HPHT) process without metal catalysts, based on mixtures of naphthalene (C_10_H_8_) and tetrakis(trimethylsilyl)silane (C_12_H_36_Si_5_) with different silicon-to-carbon (Si/C) ratios in the initial compound. In this work, we focus on two samples, namely sample A (Si/C ratio: 0.008) and sample B (Si/C ratio: 0.05); the remaining samples did not show clean SiV spectral signatures, and are discussed in the Supplementary Information. HPHT treatment of the initial homogeneous mixtures was carried out in a high-pressure apparatus of “Toroid” type^21^. The experimental procedure consists of loading the high-pressure apparatus to 8.0 GPa, heating the samples up to 1300 °C and 1450 °C for samples A and B, respectively, and short (5 s) isothermal exposures at these temperatures.

The obtained diamond products in both samples consist of nano- and submicron-sized diamond fractions, but with different particle size distributions. As we are primarily interested in small NDs, we investigate only the smallest size fraction from each sample, which consists of NDs 10–30 nm in size for sample A, and 50–100 nm for sample B. The difference in ND size distributions can be attributed to the higher growth temperature inducing a more active cumulative recrystallisation process for sample B, which leads to larger NDs. This has also been observed in carbon nanosystems where a hydrocarbon component is introduced^22^.

In contrast to NDs grown via chemical vapor deposition (CVD) on a silicon or metal substrate^9,11^, these samples are produced in a powder form, making them convenient for further processing and subsequent spatial manipulation, which is crucial for coupling to photonic nanostructures.

After extraction from the high-pressure apparatus, both samples undergo ultrasonication and centrifugation to reduce clustering and to isolate the smallest NDs, respectively, before being spin-coated onto a silicon substrate for further characterisation (see Supplementary Information for detailed description of sample preparation).

**Figure 1:**
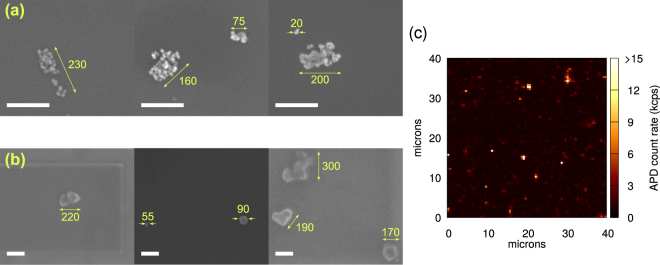
(**a**,**b**) SEM pictures of samples A and B, respectively. Numbers show approximate sizes (in nm) of individual nanodiamonds or clusters. White lines are scale bars of 200 nm length. (**c**) Typical confocal scan map with 0.5 *μ*m step size. Isolated bright spots indicate fluorescent emitters.

Despite the ultrasonication, we are unable to eliminate clustering completely; a similar issue was reported in ref.^10^ with HPHT NDs. Some SEM images of the samples are shown in Fig. 1a and b, revealing individual NDs and isolated clusters of up to ~300 nm in size for both samples.

## Experimental Setup

The optical properties of the NDs are studied with a home-built confocal microscope at room temperature (see Supplementary Information for detailed description). Briefly, off-resonant excitation light from a continuous-wave 532 nm laser is focused onto the sample through an air objective (NA = 0.95). The emission passes through a notch filter to reject the excitation light, is collected into a single-mode fiber, and directed to either a grating spectrometer for photoluminescence (PL) measurements, or to a Hanbury-Brown and Twiss (HBT) interferometer for measurements of the *g*^(2)^ second-order correlation function using single photon counters. Except for spectrometer measurements, the emission is additionally filtered by a 740 nm narrowband filter (Semrock, bandwith 13 nm).

## Results and Discussion

A typical confocal scan map of a sample is shown in Fig. 1c. Isolated bright spots reveal possible SiV candidates that would be confirmed by PL measurements; the fraction of bright spots that show a SiV spectral signature is ~30% for sample A and ~50% for sample B (see Supplementary Information). We note that it in our setup, it is not possible to resolve multiple emitters in a ND crystal or cluster within the diffraction-limited confocal detection spot. We then measure the *g*^(2)^ function of SiV centres to determine if they are single emitters, and additionally analyze the polarisation and saturation behavior of single emitters.

### Photostability

Many emitters from sample A are not stable, and 33 out of 53 emitters containing SiV centres suffered from either photobleaching or blinking under continuous excitation over a few minutes, even under low excitation power below 300 *μ*W. Blinking refers to intermittent fluorescence alternating between on/off states, while photobleaching refers to a gradual but permanent loss of fluorescence that does not recover even after long waiting times. In contrast, all 40 investigated emitters from sample B are stable for >30 mins, even under higher excitation powers of above 4 mW.

The lack of photostability has been studied for various types of quantum emitters^23,24^. For colour centres in NDs, possible mechanisms inlcude charge state switching (photoionisation) and the capture of electrons in surface traps^8^. Photoionization might be more pronounced in smaller NDs due to a lack of excess electrons^25^, and is a possible reason for the increased stability of sample B over sample A; Bradac *et al*.^26^ also report blinking behaviour of nitrogen-vacancy centres in small (5 nm) discrete NDs not seen in larger aggregates. Moreover, the better surface quality of larger NDs in our samples may indicate the presence of fewer surface defects acting as electron traps, thus contributing to increased photostability (see Supplementary Information). Further surface treatment may improve photostability further^26,27^.

### Photoluminiscence spectra

**Figure 2:**
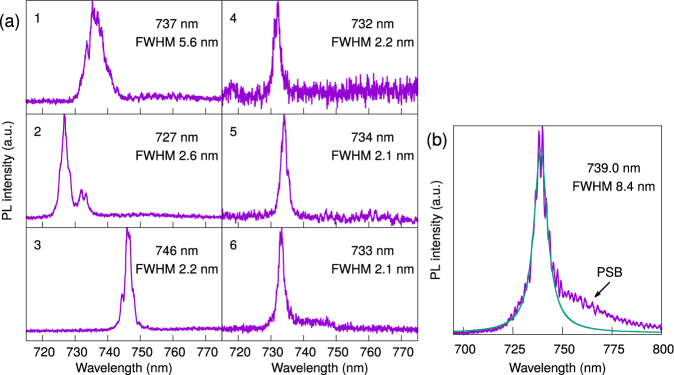
(**a**) Photoluminiscence (PL) spectra of single SiV centres in sample A, labelled as emitters 1–6. The central wavelengths of the zero-phonon line (ZPL) peak and the full-width at half-maximum (FWHM) are derived from a Lorentzian fit. (**b**) Averaged PL spectrum of 12 fluorescent spots in sample B, overlaid with the Lorentzian fit used to measure the overall linewidth. The phonon sideband (PSB) above 750 nm is clearly visible.

PL measurements of stable emitters in sample A revealed fluorescence peaks scattered around the nominal ZPL wavelength of 737 nm. Similar observations for SiV centres in NDs were reported elsewhere, and were attributed to local strain effects in smaller NDs^9,16,28,29^. Here, we identify fluorescence peaks within the range of 737 ± 10 nm as SiV centres; a few other peaks were observed at >750 nm, but these were rejected. Most of the peaks do not have a distinct phonon sideband (PSB), and from Lorentzian fits we obtain full-width at half-maximum (FWHM) values of 2.1–2.9 nm, except for two emitters with a FWHM of 3.9 nm and 5.6 nm. The spectra of six emitters labelled 1–6, later confirmed to be single SiV centres, are shown in Fig. 2a.

In contrast, the emitters in sample B showed almost identical PL spectra, with a clearly visible PSB. Figure 2b shows an averaged spectrum of 12 fluorescent spots (all but one contain multiple SiV centres), with a central ZPL peak of 739.0 nm and a FWHM of 8.4 nm. The sole single emitter showed a FWHM of 7.8 nm. Here, we cannot distinguish if the large linewidth is caused by a broad inhomogenous distribution of ZPL peaks, or if individual SiV centres have a broad ZPL spectrum.

### *g*^(2)^ function

To identify if a fluorescent spot consists of a single SiV centre instead of multiple emitters, we consider the *g*^(2)^ function between the two output detectors of the HBT interferometer. We do not perform any background corrections, and fit the data to a realistic model as follows^8^. The *g*^(2)^ function of an ideal three-level system is given by

1 $${g}^{\mathrm{(2)}}(\tau )=1-\mathrm{(1}+\alpha )\exp (-|\tau |/{\tau }_{1})+\alpha \,\exp (-|\tau |/{\tau }_{2}),$$

where *τ*_1_ and *τ*_2_ are the antibunching and bunching time constants, respectively, and *α* describes the degree of bunching. The effect of background noise can be described by ${\rho }^{2}=\frac{{S}^{2}}{{(S+B)}^{2}}$, where *S* and *B* are signal and background intensities, respectively, yielding

2 $${g}_{{\rm{noisy}}}^{(2)}(\tau )=1+{\rho }^{2}({g}^{(2)}(\tau )-1).$$

Equation (2) is then convolved with the independently measured timing response function of the setup, which is well-approximated by a Gaussian with *σ* ~ 0.5 ns (see Supplementary Information for details). The final expression is used for fitting to the measured data.

**Figure 3:**
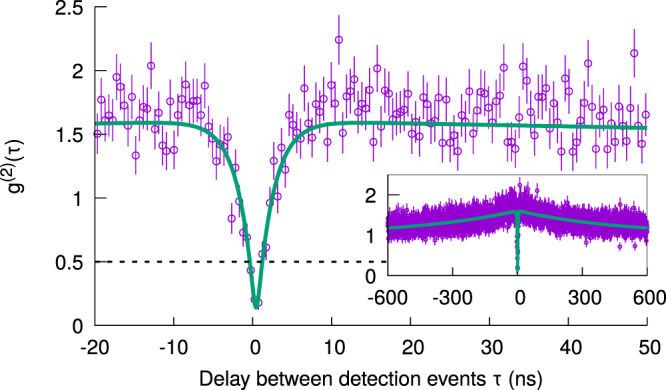
Measured *g*^(2)^ function of fluorescence from an emitter, with an antibunching dip of *g*^(2)^(0) < 0.5 confirming that it is a single SiV centre. The solid line is a fit to a three-level model that accounts for detector timing jitter and background noise. Inset plots the same measurement at a longer time scale to show the bunching behavior.

Only emitters with the characteristic antibunching signal of *g*^(2)^(0) < 0.5 can be clearly identified as single SiV centres (see Fig. 3). For sample A, 6 out of 20 stable emitters show *g*^(2)^(0) < 0.5 (30%), while we observe only 1 single emitter out of 40 candidates (2.5%) for sample B.

Although the sizes of individual NDs in sample A are smaller than in sample B, we observe isolated clusters of similar sizes in both samples (Fig. 1a,b). As such, for each investigated emitter, we regard the volume of NDs within the confocal microscope detection spot as approximately equal for both samples, and attribute the difference in SiV centre abundance of both samples to the relative silicon content (i.e. Si/C ratio) in the initial growth compound. We conclude that the lower Si/C ratio for sample A has increased the proportion of SiV-containing NDs that hosts only a single emitter. Thus, tuning the growth conditions of NDs can aid the production of NDs with an optimised abundance of SiV centres, depending on the intended application.

From the *g*^(2)^ fits, we are also able to extract the values of *τ*_1_, which ranges from 0.9 ± 0.2 ns to 3.8 ± 0.2 ns for all the investigated single emitters. The range of values is comparable to other reported values of *τ*_1_ for SiV centres in NDs^10,11^.

### Fluorescence polarisation of single emitters

We then analyze the polarisation of the emitted fluorescence of the single SiV centres by placing a polarizer after the dichroic beamsplitter, and measuring the fluorescence count rate *I* as a function of the rotation angle of the polarizer. The emission of SiV centres is known to be linearly polarised^28^, and the polarisation contrast can be described by the visibility

3 $$V=\frac{{I}_{{\rm{\max }}}-{I}_{{\rm{\min }}}}{{I}_{{\rm{\max }}}+{I}_{{\rm{\min }}}}\mathrm{.}$$

**Figure 4:**
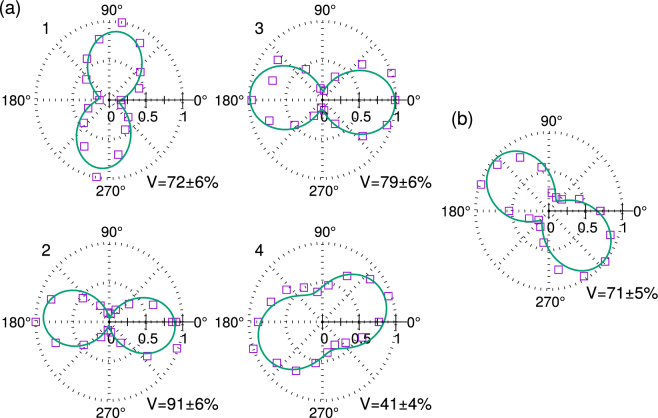
Normalised polar plots of fluorescence count rate *I* of (**a**) single emitters 1–4 from sample A and (**b**) the single emitter from sample B, as a function of the rotation angle *θ* of a polariser placed after the dichroic beamsplitter. Solid lines show fits to a cos^2^*θ* model, from which we obtain the visib1ility *V* = (*I*_max_ − *I*_min_)/(*I*_max_ + *I*_min_).

Unfortunately, emitters 5 and 6 from sample A were bleached during this measurement. The results for the other single emitters are shown in Fig. 4.

Except for one of the single emitters from sample A, the visibility *V* is fairly high at >70%. The lack of full visibility can be attributed to several reasons. The dichroic beamsplitter induces polarisation changes in the transmitted light, and although we correct for the polarisation-dependent transmission, the dichroic beamsplitter causes an additional loss of linear polarisation of ~10%^28^. Besides, polarisation anisotrpy due to imaging from a high NA objective^28,30^, background luminescence from the diamond material, and contributions from another distant, weakly excited emitter can also degrade polarisation contrast. As such, in applications where high visibility is critical, a polariser can be used to project the fluorescence onto an optimal linear polarisation.

### Saturation behavior of single emitters

**Figure 5:**
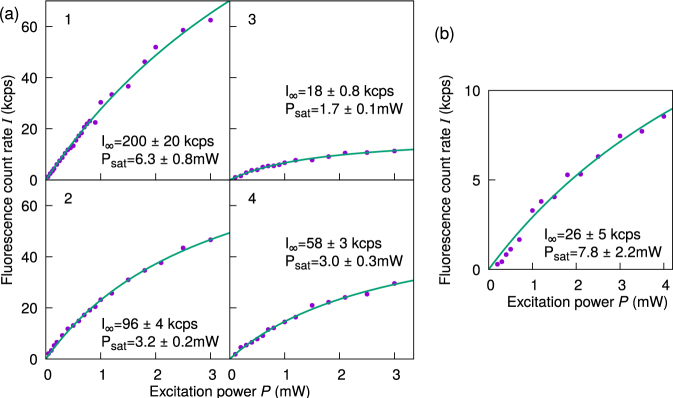
Fluorescence count rate *I* of (**a**) single emitters 1–4 from sample A and (**b**) the single emitter from sample B, as a function of the power *P* of the 532 nm excitation light. The data is corrected for background counts. Solid lines represent fits to the function $I={I}_{\infty }\frac{P}{P+{P}_{{\rm{sat}}}}$, from which we extract the maximum count rate *I*_∞_ and saturation power *P*_sat_. The poor fit leading to a large uncertainty in *I*_∞_ and *P*_sat_ in (**b**) might be due to an onset of photobleaching at higher excitation powers.

The saturation behavior of the single emitters can be described by the equation

4 $$I={I}_{\infty }\frac{P}{P+{P}_{{\rm{sat}}}},$$

where *I*_∞_ is the maximum fluorescence count rate, *P* is the excitation power, and *P*_sat_ is the saturation power. In our measurements, we first maximise *I* by rotating a half-wave plate in the excitation beam path, then recording *I* as a function of the excitation beam power (see Fig. 5). The data is corrected for the background count rate measured at a nearby spot on the substrate without any fluorescent NDs, then fitted to equation (4).

The observed saturation behavior varied greatly between the single emitters, with the fitted *I*_∞_ values ranging from 18 kcps to 200 kcps, and *P*_sat_ ranging from 1.7 ± 0.1 mW to 7.8 ± 2.2 mW. We note that we were not able to fully observe the fluorescence above *P*_sat_ due to the onset of photobleaching at higher excitation powers, which might have caused the poor fit leading to a large uncertainty in *I*_∞_ and *P*_sat_ for the single emitter from sample B (see Fig. 5b). A choice of a longer excitation wavelength or resonant excitation could have allowed for more efficient excitation and a lower *P*_sat_^11,28^.

## Conclusion

In conclusion, we have characterised SiV centres in HPHT ND samples. The lower growth temperature for sample A has led to smaller ND sizes compared to sample B. For sample A which is obtained from a mixture with low silicon content (Si/C ratio: 0.008), among NDs that show a SiV spectral signature, we observe a 30% fraction (6 out of 20 candidates) of NDs that contain a single emitter. This is roughly ten times higher than in sample B which is obtained from a mixture with high silicon content (Si/C ratio: 0.05), where the corresponding fraction is 2.5% (1 out of 40 candidates). We summarise the observations of all the single emitters in Table 1. Our results demonstrate that varying the synthesis parameters and the doping impurity content in the initial growth compound can effectively influence the ND size distribution and the abundance of single photon emitters. This opens up possibilities for targeted synthesis of diamond materials for different applications.

**Table 1:** Summary of the observed single SiV centres. We were not able to measure the polarisation and saturation behavior of emitters 5 and 6 from sample 1.

Sample/emitter	ZPL/FWHM (nm)	*V* (%)	*I*_∞_ (kcps)	*P*_sat_ (mW)
A/1	737/5.6	72 ± 6	200 ± 20	6.3 ± 0.8
A/2	727/2.6	91 ± 6	96 ± 4	3.2 ± 0.2
A/3	746/2.2	79 ± 6	18 ± 0.8	1.7 ± 0.1
A/4	732/2.2	41 ± 4	58 ± 3	3.0 ± 0.3
A/5	734/2.1	−	−	−
A/6	733/2.1	−	−	−
B/1	739/7.8	71 ± 5	26 ± 5	7.8 ± 2.2

### Data availability

The data that support the findings of this study are available from the corresponding author upon reasonable request.

## Electronic supplementary material

Supplementary Information
