# Supplementary material for: Varying temperature and silicon content in nanodiamond growth: effects on silicon-vacancy centres
Source: Sci Rep. 2018 Feb 28;8:3792. doi: 10.1038/s41598-018-21953-2 (PMC5830582; doi:10.1038/s41598-018-21953-2)
Supplement: Supplementary file 1 — Supplementary Information [file 41598_2018_21953_MOESM1_ESM.pdf]

Supplementary Information for:
Varying temperature and silicon content in nanodiamond growth: effects on silicon-vacancy centers

Sumin Choi[1,†,*], Victor Leong[1,†], Valery A. Davydov[2], Viatcheslav N. Agafonov[3], Marcus W.O. Cheong[1], Dmitry A. Kalashnikov[1], and Leonid A. Krivitsky[1]

[1]Data Storage Institute, Agency for Science, Technology and Research, 138634 Singapore
[2]L.F. Vereshchagin Institute for High Pressure Physics, the Russian Academy of Sciences, Troitsk, Moscow, 142190 Russia
[3]GREMAN, UMR CNRS-7347, Université F. Rabelais, 37200 Tours, France
[†]These authors contributed equally to this work.
[*]choism@dsi.a-star.edu.sg

S1 Sample preparation

We study several nanodiamond (ND) samples grown using a HPHT process under varying conditions (see Table S1). The PL spectra of samples C, D and E do not show a clean SiV signature, but instead contain contributions from both SiV and nitrogen-vacancy (NV) centers. A typical example is shown in Figure S1. It appears that the longer isothermal exposures for these samples resulted in a much higher abundance of NV centers compared to samples A and B, and thus we are unable to obtain fluorescence that is emitted only from SiV centers. Therefore, samples C, D and E were not studied further in detail, and the remainder of this work focuses on samples A and B.

Samples A and B are prepared differently after extraction from the high-pressure apparatus. Sample A is boiled with a mixture of sulfuric, hydrochloric, and nitric aicds for 4 h at 150°C to remove the graphite content. After washing with distilled water, the sample is centrifuged (5000 g 10 min) in 100% ethanol, then the supernatant is extracted and centrifuged again. The precipitate of the second centrifugation is suspended in isopropanol and ultrasonicated for 30 mins. 10 µl of the sample suspension is then spin-coated onto a silicon substrate.

There is only a small amount of NDs in sample B; it is not treated with acid boiling to prevent excessive loss of NDs. Instead, it is suspended in isopropanol, ultrasonicated for 30 mins, then centrifuged (1900 g 60 min). The top portion (3 ml out of 12 ml) is extracted, and 10 µl of the sample suspension is spin-coated onto a silicon substrate.

Table S1. Growth conditions for the different nanodiamond samples, detailing the Si/C ratio of the initial compound, pressure, and the temperature and duation of isothermal exposure.

Sample	Si/C ratio	Pressure	Isothermal exposure
A	0.008	8.0 GPa	1300°C, 5 s
B	0.05	8.0 GPa	1450°C, 5 s
C	0.05	8.0 GPa	1300°C, 15 s
D	0.08	8.0 GPa	1300°C, 15 s
E	0.08	8.0 GPa	1300°C, 20 s

S2 Surface effects of nanodiamonds on photostability

The dependence of colour centre photostability in NDs on surface effects has been discussed in the literature[1–3]. The treatment of our ND samples with acids cause -COOH groups to be induced on the ND surface, and we confirm this with IR and XPS measurements. This agrees with similar studies in the literature[4]. However, a definitive answer of the influence of these groups on the photostability will require further detailed work, including the considerations of chemistry and structural defects in the NDs.

Scanning electron microscope (SEM) images also show a more perfect surface structure for larger NDs in our samples (see Fig. S2). This may indicate a lower number of surface defects acting as electron traps in larger NDs. Even though there is no direct experimental evidence of this effect, it may serve as an additional argument to support the observation of increased photostability of larger NDs.

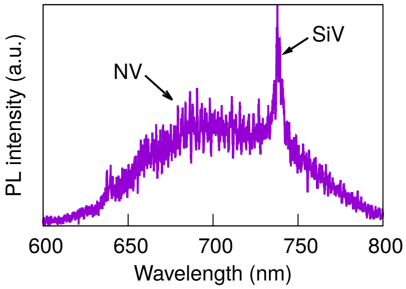

Figure S1. Typical PL spectrum from samples C, D and E, showing spectral signatures of both SiV centers (ZPL at 737 nm) and nitrogen-vacancy (NV) centers (ZPL at 638 nm, broad phonon sideband).

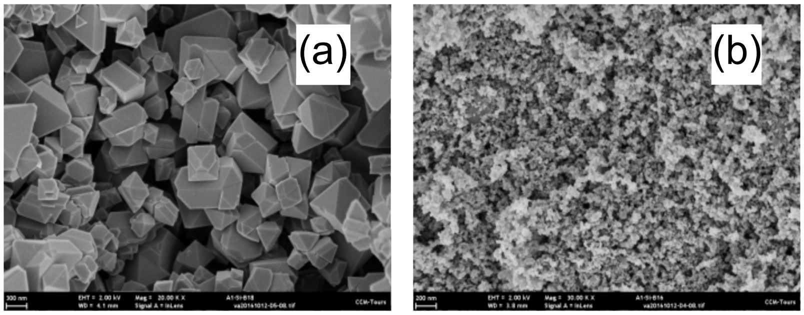

Figure S2. SEM image of (a) sub-micron-sized and (b) nano-sized NDs grown using the HPHT process at 8.0 GPa.

S3 Experimental setup

Optical characterization is performed with a home-built room-temperature confocal microscope (see Fig. S3). The sample is mounted on a 3D piezoelectric stage (Piezosystem Jena, TRITOR 100 SG), which is scanned in 0.5 µm steps. Off-resonant excitation light from a continuous-wave 532 nm laser (Oxxius) is focused onto the sample through an air objective (Nikon, NA=0.95). A 532 nm half-wave plate in the excitation arm is rotated to best align the polarization of the excitation laser to the SiV dipole orientation, and thus maximize the amount of fluorescence. The emission is filtered by a dichroic mirror (Semrock FF555-Di03), a notch filter (Semrock NF03-532E), and a narrowband filter (Semrock FF01-740/13); these filters reject the excitation light and transmit only in the vicinity of the ZPL. We choose to collect the emission into a single-mode fiber (Thorlabs SM600) to minimize the collection of background fluorescence.

Photoluminescence (PL) measurements are performed with a grating spectrometer (Princeton Instruments IsoPlane 160, 0.07 nm resolution) with the narrowband filter removed. All other measurements are performed with the Hanbury-Brown and Twiss (HBT) interferometer, which consists of a 50/50 non-polarizing beamsplitter with two avalanche photodetectors (APDs,

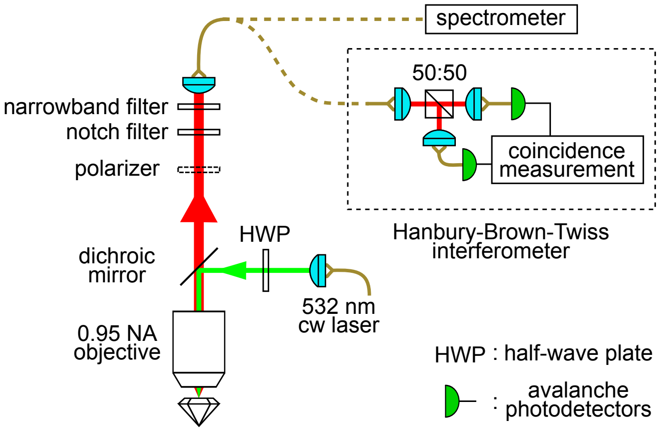

Figure S3. Sketch of the experimental setup.

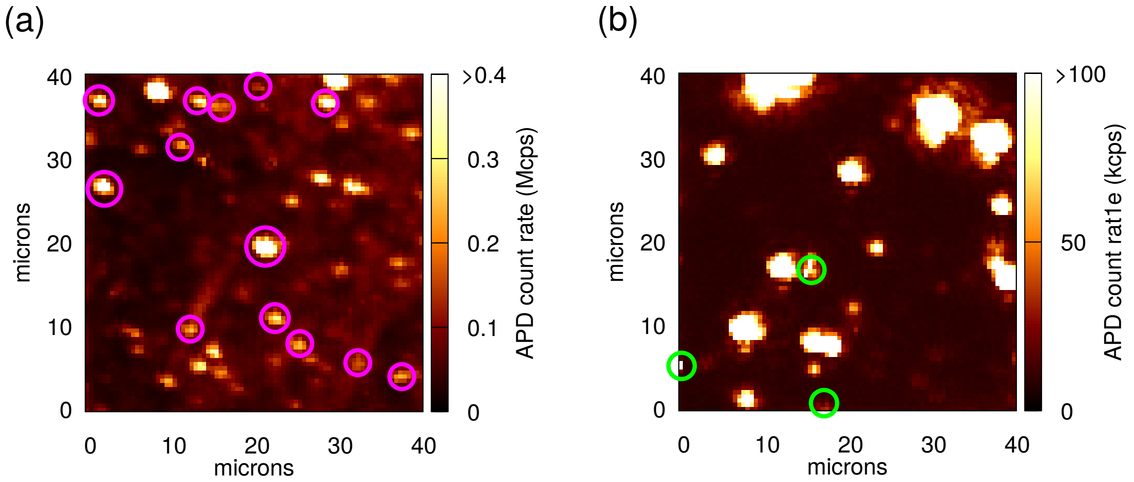

Figure S4. Typical confocal scan images taken without the narrowband filter. (a) Sample A. Marked circles show bright spots with SiV spectral signatures. (b) Sample B. Marked circles show bright spots without SiV spectral signatures.

Perkin Elmer SPCM-AQRH-15) at its outputs. The reported count rates are a sum of the signals from both APDs; the APD signals are also timestamped (qutools quTAU) and analyzed to obtain the $g^{(2)}$ function. For polarization measurements, a polarizer (Thorlabs LPVIS050-MP2) is placed in the emission arm and rotated while monitoring the APD count rates.

S4 Proportion of nanodiamonds containing SiV centres

To estimate the proportion of NDs that do not contain SiV centres, we perform confocal scans of the ND samples without the narrowband filter. Typical scan images are shown in Fig. S4. Here, even NDs without emitting centres will show up due to broadband luminsecence under excitation from the 532 nm laser. We consider only bright spots not larger than several pixels (pixel size 0.5x0.5 µm), as only these would correspond to isolated NDs or small ND clusters as discussed in the main paper. Under PL measurements, the proportion of these spots that show a SiV spectral signature is ∼30% for sample A and ∼50% for sample B. We note that even with the narrowband filter, not all bright spots in the confocal scan image correspond to NDs with SiV centres, and PL measurements of their emission spectra is needed.

This estimate may not be very accurate as it is difficult to identify if a dim spot on the scan image is due to background noise or a weakly luminescent ND (or cluster). Besides, we cannot tell if a bright spot is due to one isolated ND, or a cluster of several NDs where only one of them contain a SiV centre. As such, from the proportion of SiV-containing bright spots in the confocal scan image, we are unable to directly infer the proportion of individual NDs that contain SiV centres. A better alternative might be to directly compare confocal scan images with SEM images of the same sample area.

S5 Considerations of timing jitter

The measured $g^{(2)}$ function is a convolution of the actual photon statistics of the ND fluorescence and the timing response of the setup. As the timing jitter of each APD (350 ps, from the datasheet) is already comparable to the excited state lifetime (∼1 ns), this would be a non-negligible contribution to the measurement results.

We explicitly measure the timing response of the HBT setup by using attenuated 810 nm femtosecond laser pulses as an input. As the width of the laser pulses is negligible compared to the APD jitter, the width of the peaks in the coincidences between the APDs (see Fig. S5) is a direct measure of the overall timing response of the setup. The coincidence peaks are well-approximated by a Gaussian distribution function with a standard deviation $\sigma = 493 \pm 1$ ps, obtained by averaging the fits results of multiple peaks.

The full analytical expression used to fit the $g^{(2)}$ data is a convolution of $g^{(2)}_{\text{noisy}}(\tau)$ (defined in main text) with a Gaussian function, yielding:

$$g^{(2)}_{\text{fit}}(\tau) = 1 - \frac{\rho^2}{2}\left[(1+\alpha)f(\tau,\tau_1) - \alpha f(\tau,\tau_2)\right] \tag{1}$$

where

$$f(\tau,\tau_{1,2}) = \exp\left(\frac{\sigma^2 - 2\tau\tau_{1,2}}{2\tau_{1,2}^2}\right)\text{Erfc}\left(\frac{\sigma/\tau_{1,2} - \tau/\sigma}{\sqrt{2}}\right) + \exp\left(\frac{\sigma^2 + 2\tau\tau_{1,2}}{2\tau_{1,2}^2}\right)\text{Erfc}\left(\frac{\sigma/\tau_{1,2} + \tau/\sigma}{\sqrt{2}}\right) \tag{2}$$

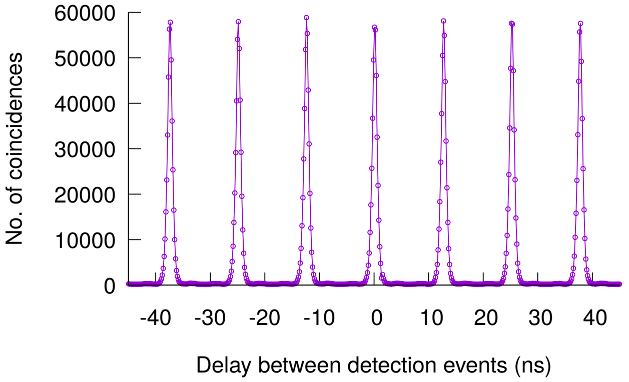

Figure S5. Measured coincidences between the two APDs of the HBT setup, with 810 nm femtosecond laser pulses as the input. The data is sorted into 162 ps wide time bins.

and Erfc is the complementary error function.

References

1. Neu, E., Agio, M. & Becher, C. Photophysics of single silicon vacancy centers in diamond: implications for single photon emission. *Opt. Express* **20**, 19956–19971 (2012).

2. Bradac, C. *et al.* Observation and control of blinking nitrogen-vacancy centres in discrete nanodiamonds. *Nat. Nanotechnol.* **5**, 345–349 (2010).

3. Cui, S. & Hu, E. L. Increased negatively charged nitrogen-vacancy centers in fluorinated diamond. *Appl. Phys. Lett.* **103**, 051603 (2013).

4. Davydov, V. *et al.* Solid state synthesis of carbon-encapsulated iron carbide nanoparticles and their interaction with living cells. *J. Mater. Chem. B* **2**, 4250–4261 (2014).
